# Ice Houses

**Published:** 1888-06

**Authors:** 


### ICE HOUSES.

*The American Architect* advises as follows :

1. The ice house floor should be above the level of the gronnd, or, at least, should be sufficiently above some neighboring area to give an outfall for a drain, constructed in such a way as to keep the floor clear of standing water.

2. The walls should be hollow. A four inch lining wall, tied to the outer wall with hoop iron, and with a three inch air space, would answer ; but it would be better, if the air space is thoroughly drained, to fill it with mineral wool, or some similar substance, to prevent the movement of the air entangled in the fibers, and thus check the transference by convection of heat from the outside to the lining wall.

3. A roof of thick plank will keep out heat far better than one of thin boards with an air space under it.

4. Shingles will be much better for roofing than slate.

5. It is best to ventilate the upper portion of the building. If no ventilation is provided, the confined air under the roof becomes intensely heated in summer ; and outlets should be provided, at the highest part with inlets at convenient points, to keep the temperature of the air over the ice at least down to that of the exterior atmosphere.

